# Luteinizing hormone-releasing hormone agonists versus orchiectomy in the treatment of prostate cancer: A systematic review

**DOI:** 10.3389/fendo.2023.1131715

**Published:** 2023-02-06

**Authors:** Xianlu Zhang, Gejun Zhang, Jianfeng Wang, Yanli Wang

**Affiliations:** ^1^ Department of Urology Surgery, The First Affiliated Hospital of China Medical University, Shenyang, Liaoning, China; ^2^ Department of Infectious Diseases, The First Affiliated Hospital of China Medical University, Shenyang, China

**Keywords:** luteinizing hormone-releasing hormone agonists, orchiectomy, prostate cancer, androgen deprivation therapy, meta-analysis

## Abstract

**Background:**

Orchiectomy has been replaced by medication represented by luteinizing hormone-releasing hormone (LHRH) agonist as the first-line therapy for androgen deprivation therapy (ADT). After the wide application of LHRH agonist, the side-effects of long-term ADT were noticed. It is time to reconsider the role of medication and surgeries in the treatment of prostate cancer.

**Methods:**

Embase, Pubmed, Web of science and Cochrane library were searched for relevant trials. Quality of the studies and risk of bias were assessed by using the Newcastle-Ottawa Scale (NOS). Therapeutic and adverse effects, as well as long-term metabolic adverse effects were extracted from the selected studies. The data synthesized in meta-analyses were performed with R software (4.2.1). Risk ratio (RR) with its 95% confidence interval (CI) was calculated by combining outcome data including complete and partial response rate, progression rate, death rate and adverse effects such as hot flash and increase in pain. Descriptive analysis was performed among the prostate specific antigen (PSA), testosterone and metabolic adverse effects due to a lack of homogeneity of frailty measures.

**Results:**

1,711 participants from 11 studies were included in our systematic review. 1,258 patients from six studies were included in the meta-analysis. Based on the meta-analysis, the therapeutic and adverse outcomes included overall response rate, complete response rate, partial response rate, stable rate, progression rate, death rate and hot flashes. No statistical significance was observed between LHRH agonists and orchiectomy. Compared with surgery, LHRH agonist elevated the risk of the increase in pain. In descriptive analysis, it was shown that the therapeutic effects between PSA and testosterone also showed no significant difference. Both groups had lipid and glucose metabolic disorders, and a few studies reported worse lipid metabolic performance in orchiectomy group and worse insulin resistance in LHRH agonist group.

**Conclusion:**

We found that the therapeutic outcomes were similar between the two options. The results of lipid and glucose metabolic abnormality were controversial in existing studies. The direct comparison studies on metabolic adverse effects should be performed in the future. The therapeutic, metabolic, psychological and economical effects should be considered before applying ADT methods.

**Systematic review registration:**

https://www.crd.york.ac.uk/prospero/, identifier CRD42022365891.

## 1 Introduction

Androgen deprivation therapy (ADT) has become the first-line therapy for advanced prostate cancer (PCa) since the relationship between prostate cancer and hormonal dependence was interpreted. Before medication approach was developed, castration was achieved by surgical approach (1). With similar response rates, 1-year survival and adverse effects, LHRH agonists represented by goserelin have been widely used as a substitute of surgeries for 2 decades (2). The long-term adverse effects related with cardiovascular system and glucose tolerance were noticed by investigators. This systematic review aimed to compare the therapeutic and adverse effects of the two options review the metabolic adverse effects (AEs) of medical endocrine therapy reported in the latest studies, and reconsider the role of medication and surgeries in ADT for prostate cancer.

## 2 Methods

This systematic review was conducted following the PRISMA statement (3) and was registered on PROSPERO. (Registration No. CRD42022365891) (4). The PRISMA checklist is provided in **Additional file 4**.

### 2.1 Information sources and search strategy

PubMed, Embase, Web of Science and Cochrane library were searched for Randomized Controlled Trials (RCTs) or cohort studies comparing the therapeutic effects and AEs between the LHRH agonist and orchiectomy from inception to October 1, 2022. Two investigators independently reviewed all titles and abstracts. Subject terms mainly included ‘Prostate cancer’, ‘Luteinizing Hormone-Releasing Hormone Agonists’ and ‘orchiectomy’. Detailed search strategy is provided in **Additional file 1**.

### 2.2 Inclusion and exclusion criteria

Studies meeting the following criteria were included:

Types of Participants (P): Patients diagnosed with prostate cancer without previous ADT history.
Types of interventions (I): Patients treated with LHRH agonist.
Types of comparisons (C): Patients treated with orchiectomy.
Outcome measures (O): Therapeutic effects: PSA, serum testosterone, objective response rate and other therapeutic measurements. Adverse effects: hot flash, increase in pain, lipid or glucose metabolic AEs.
Types of Study(S): Published RCTs or cohort studies.

Literature review, animal study, conference summary, repeated publication, studies not in English, and studies with incomplete or unavailable data were excluded.

### 2.3 Data extraction and quality assessment

Two reviewers extracted data by using a pre-designed form which included first author, publication date, nation, baseline characteristics of participants (mean age, gender, body mass index, etc.), grouping, and treatment to patients. Cohort studies were assessed using Newcastle-Ottawa Scale (NOS). Funnel plots was created to visually assess the degree of publication bias and its effect on the study finding. The NOS and funnel plots are provided in **Supplementary material 2**. Data extraction and quality assessment were conducted by two reviewers independently. Disagreements were resolved *via* discussion or by consulting a third reviewer.

### 2.4 Data synthesis

A cumulative meta-analysis was conducted using Meta package of R software (version 4.1.2). Weighted mean difference was used to compare continuous variables and odd ratio. Risk ratio and hazard ratio were used to compare dichotomous variables, respectively. All results were reported with 95% CIs. Statistical heterogeneity between studies was tested using the I² statistic. The random-effects model was applied if there was significant heterogeneity among the included studies (I² > 50%), otherwise (I² <50%), the fixed-effects model was adopted. Forest plots were also created. P < 0.05 indicated a statistically significant effect. The original data and R code are presented in **Additional file 3**.

## 3 Results

### 3.1 Characteristics of included studies

**Figure 1:**
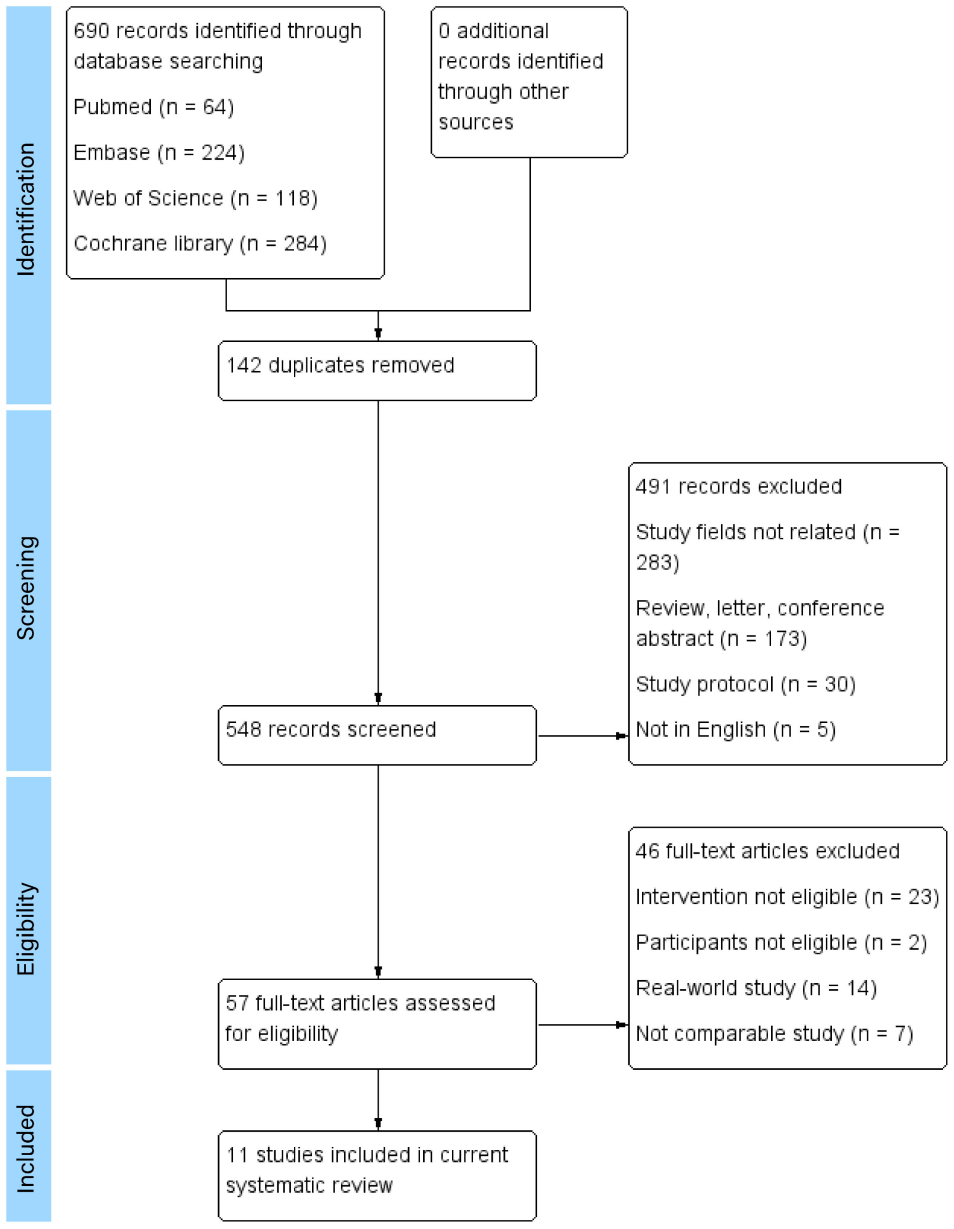
Study selection process and the reasons for exclusion.

A total of 690 articles were found and assessed according to inclusion criteria (64 from Pubmed, 224 from Embase, 118 from Web of Science and 284 from the Cochrane library). The titles, abstracts and the full texts of the articles were reviewed. Articles were selected based on inclusion and exclusion criteria. The references of selected articles were searched for further eligible articles. There were 11 articles involving 1,711 participants included in the systematic review (5–15). The study selection process and the reasons for exclusion are shown in **Figure 1**. The characteristics of the included studies are shown in **Table 1**.

**Table 1:** Characteristics of included studies.

Study	Year	Country	Patients	Sample size		outcomes
				Orchiectomy	LHRH agonist	
Agarwala et al. (5)	2021	India	metastatic hormone- sensitive prostate cancer (mHSPC)	51	54	Mean nadir PSA level; Mean time to progression
Kaisary et al. (6)	1991	UK	Metastatic PCa	144	148	Response rate
Parmar et al. (9)	1985	USA	Metastatic PCa	32	58	Response rate
Parmar et al. (10)	1987	USA	Metastatic PCa	49	55	Response rate; Testosterone
Ryan et al. (11)	1988	UK	Metastatic PCa	163	162	Response rate
Selvi et al. (12)	2019	Turkey	mHSPC	129	60	PSA level; Testosterone level; emotional assessments
Soloway et al. (13)	1991	USA	PCa	83	81	Response rate
Vargas et al. (14)	2016	Brazil	PCa	46	56	TC; LDL cholesterol; Triglyceride; Hemoglobin; Fast glucose; HOMA-IR
Vogelzang et al. (15)	1995	USA	PCa	145	138	Response rate
Østergren et al. (7)	2016	USA	PCa without curative options	28	29	Testosterone level; DHEAS level
Østergren et al. (8)	2019	USA	PCa without curative options	28	29	Fast glucose; HOMA-IR; body composition; total fat mass; VAT; FAT

This systematic review included 10 cohorts from 11 articles. All studies were prospective cohort design with sample sizes ranging from 57 to 358. The included studies were conducted in different countries, including USA (7, 8, 13, 15), UK (6, 9–11), Brazil (14), Turkey (12), and India (5). The studies could be divided into 2 groups according to publication years, with 6 published before 2000 (6, 9–11, 13, 15) and another 5 after 2015 (5, 7, 8, 12, 14).

Ten studies focused on therapeutic effects on prostate cancer, among which 6 published before 2000 reported objective response (6, 9–11, 13, 15), 2 reported PSA and testosterone decline, 1 reported nadir PSA and time to progression (TTP) (5), and 1 only reported testosterone decline (7). One of these studies also measured health and emotional status (12). The other two studies focused on metabolic AEs including lipid and glucose metabolism (8, 14).

**Figure 2:**
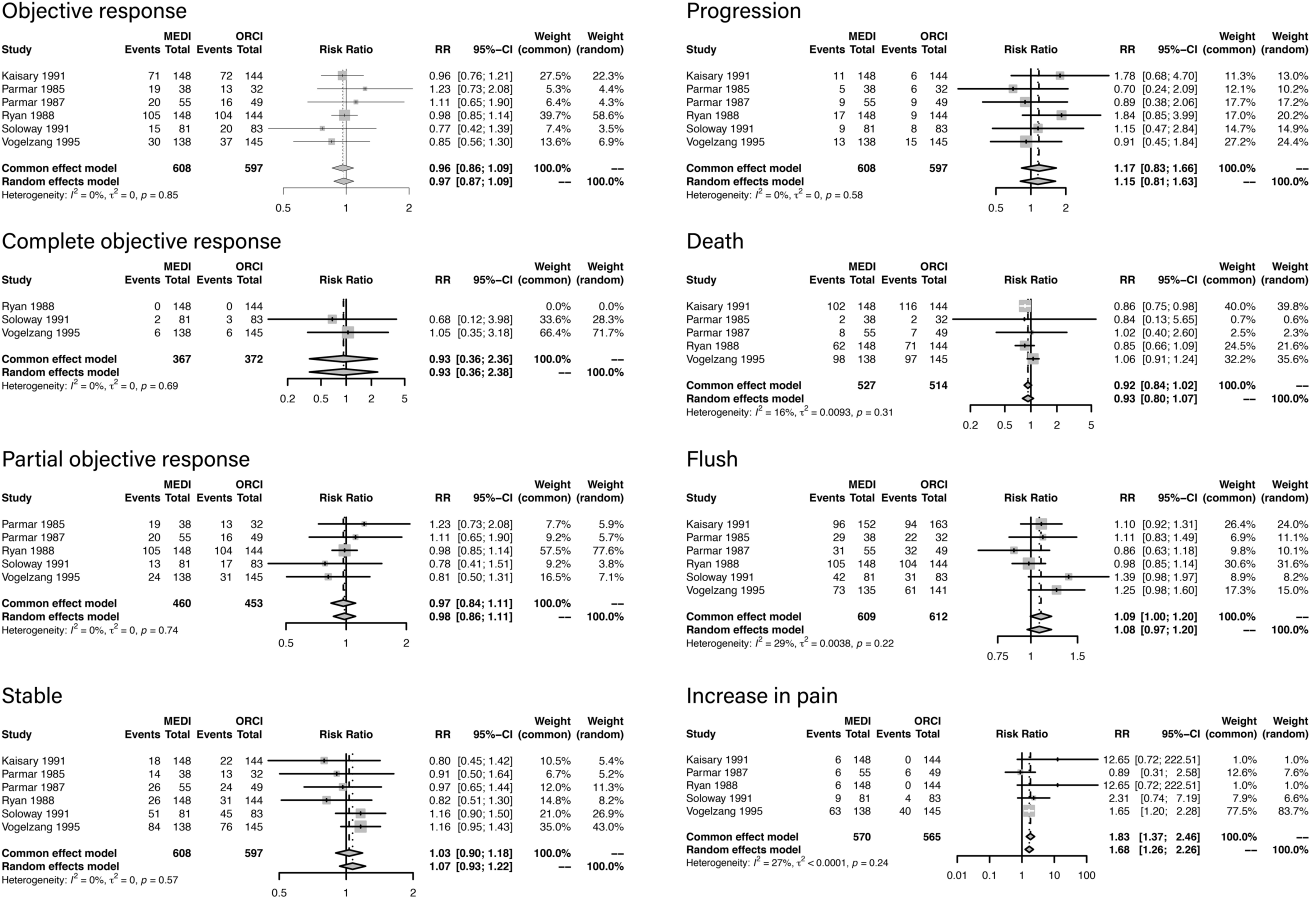
Forest plots of outcomes.

According to the characteristics of the outcomes data, a meta-analysis was conducted among the articles published before 2000 Descriptive analysis was performed among all the studies due to a lack of homogeneity of frailty measures the studies published in recent years. Among the six studies included in the meta-analysis, 642 patients were treated with LHRH agonist and 616 were treated with orchiectomy. Forest plots of outcomes are presented in **Figure 2**. The criteria of objective clinical response were displayed in **Additional file 2**.

### 3.2 Result of meta-analysis

Objective response was analyzed in six studies, and there was no difference between LHRH agonist treatment and orchiectomy (RR, 0.96; 95%CI, 0.86-1.09; P=0.85).

Complete objective response was analyzed in three studies, and there was no difference between LHRH agonist treatment and orchiectomy (RR, 0.93; 95%CI, 0.36-2.36; P=0.69).

Partial objective response was analyzed in five studies, and there was no difference between LHRH agonist treatment and orchiectomy (RR, 0.97; 95%CI, 0.84-1.11; P=0.74).

Stable status was analyzed in six studies, and there was no difference between LHRH agonist treatment and orchiectomy (RR, 1.03; 95%CI, 0.90-1.18; P=0.57).

Progression rate was analyzed in six studies, and there was no difference between LHRH agonist treatment and orchiectomy (RR, 1.17; 95%CI, 0.83-1.66; P=0.58).

Death rate was analyzed in five studies, and there was no difference between LHRH agonist treatment and orchiectomy (RR, 0.92; 95%CI, 0.84-1.02; P=0.31).

Flush rate was analyzed in six studies, and there was no difference between LHRH agonist treatment and orchiectomy (RR, 0.92; 95%CI, 0.84-1.02; P=0.22).

Increase in pain rate was analyzed in five studies. Compared with orchiectomy, LHRH agonist treatment elevated the risk of increase in pain (RR, 1.83; 95%CI, 1.37-2.46; P=0.24).

### 3.3 PSA and testosterone

PSA was evaluated in two studies. One study provided 3-month PSA, and the other provided the nadir PSA value. The mean PSA varied from 4.7 to 9.8 ng/ml. No statistical difference was observed between groups.

Testosterone was evaluated in four studies. The serum testosterone level in 3-24 months were provided by these studies. All groups reached a castration level. Among all checkpoints in each group, no significant difference was found between LHRH agonist and orchiectomy groups.

### 3.4 Metabolic side effects

Lipid metabolic side effects were evaluated in two studies. One study evaluated the total cholesterol (TC), low density lipoprotein (LDL) cholesterol and triglycerides. The other study evaluated total fat mass, subcutaneous adipose tissue volume (SAT), visceral adipose tissue volume (VAT) and body fat percentage. No significant difference was observed in TC, LDL cholesterol and triglycerides between groups, however, there was a time-dependent difference among these indicators. Additionally, mean total fat mass, SAT and increase in body fat percentage were observed in those who underwent orchiectomy compared with LHRH agonist.

Fast plasma glucose and homeostasis model assessment-insulin resistance (HOMA-IR) were measured to assess glucose metabolism in two studies. One study indicated that there was no treatment-related difference in fast plasma glucose and HOMA-IR between groups. The other study revealed that the HOMA-IR (p = 0.044) was poorer among the patients using LHRH agonists compared with the patients treated with bilateral orchiectomy (BO).

## 4 Discussion

After the wide application of medical castration, the overall trend in the utilization of surgical castration declined from 8.4% to 3.1% in one decade according to National Cancer Database. The orchiectomy has the advantage of high patients’ compliance and cost-effectiveness. A real-world study identified 33,585 patients’ data and concluded that surgical castration was preferred among patients of non-Caucasian race, with lower median income levels, without private insurance, and diagnosed at an earlier age (16). Besides, the long-term medical castration leads to long-term adverse effects, which makes patients tend to use surgical approach. After we systematically searched the eligible articles, we noticed that the studies performed in recent years evaluated the outcomes in various levels which could not be synthesized, and studies with same evaluations were included in meta-analysis.

### 4.1 Surgical castration

Efficacy of BO in prostate cancer was first reported in 1941 (17). It is a simple surgery under regional, intrathecal or spermatic cord anesthesia, with an operation time less than one hour in outpatient department. The surgical techniques included bilateral total orchiectomy (BTO) and bilateral subcapsular orchiectomy (BSO) with similar difficulties in procedures. The study by *Selvi et al.* compared the efficacy of BTO and BSO and concluded an adequate castration level, however, the patients’ emotional satisfaction was better in BSO group than BTO group (12). There were also studies suggesting that BSO showed no psychological benefit compared to BTO (18, 19). *Issa et al.* reported the epididymal sparing bilateral simple orchiectomy as an alternative surgical approach which aimed at maintaining the esthetic appearance of the scrotum and improving patient satisfaction (20).

The castration effects of BO are exact and instant. *Maatman et al.* reported a castration serum testosterone level within 2 to 6 hours (mean 3 hours) after surgery (21). The result of our meta-analysis also suggested similar effects of LHRH agonist in various therapeutic response measurements. The studies we excluded from the data synthesis also suggested similar PSA and testosterone decline between surgical and medical approach (5, 12, 22). In terms of AEs, except for minor complications such as wound infection and hematoma, severe complications were rare (23). A survey on PCa patients reported that 22% of patients preferred surgical options and 78% of patients preferred medical castration, and the main reason was the avoidance of surgery (24). The choice can be attributed to the emotional impact under the condition that surgery and medical approach had similar effects.

### 4.2 Medical castration

Most testosterone is synthesized and secreted by testis, which is controlled by pituitary gland *via* the luteinizing hormone (LH) regulated by the hypothalamic hormone LHRH. This hypothalamic–pituitary axis coordinates the androgen hormone that stimulates the PCa. The estrogen, androgen receptor inhibitors (ARIs), LHRH agonists and antagonists target on the hypothalamic-pituitary axis. Estrogen affects various hormone-related cancer such as prostate cancer and breast cancer. Estrogen was found to play a key role in some disorders which were previously considered as having no relationship with estrogen (25–28). With the fast development of endocrine medication, estrogen was excluded from first-line therapy. Bicalutamide and flutamide were representative agents of ARIs. They are widely used in combined therapy of ADT with LHRH agonists in recent years. LHRH agonist was first approved for treatment of PCa in 1986. Studies in humans indicated that treatment with LHRH agonists could result in inhibition of ovulation in women and decreased testicular steroidogenesis in men, which led to the investigation of potential uses of LHRH agonists in the treatment of androgen-dependent PCa (29). Our systematic review suggested the equivalence of therapeutic effects between LHRH agonists and orchiectomy. However, more research is required to investigate the AEs associated with orchiectomy and LHRH agonists. Main AEs include hot flashes, lipid and glucose metabolic disorder.

### 4.3 ADT related adverse effects

Hot flash is a sudden rush of warmth in the face, neck, upper chest, and back lasting for a few seconds to an hour (30), which is reported as the most common AE. The incidence of hot flash has been estimated over 50%, but most cases are mild (31). In some rare cases, severe hot flash may influence patients’ daily life. Estrogens such as diethylstilbestrol bring the relief to hot flash (32). The difference in the incidence of hot flash between LHRH agonists and orchiectomy was not observed.

Lipid metabolic disorder and insulin resistance were observed in patients receiving ADT, which likely result in the risks of diabetes, coronary artery disease, myocardial infarction and sudden death (33). Testosterone is important in regulating energy utilization and cellular metabolism, including regulation of adipogenesis. There is a significant inverse correlation between testosterone and fasting glucose and insulin levels as well as glucose intolerance (34, 35). *Galvão’s et al.* reported that after 36 weeks of ADT, there was a significant decrease in lean mass and increase in fat mass in PCa patients (36). Our study suggested that both approaches contributed to the increase of TC, LDL cholesterol, triglycerides, fat mass, body fat percentages and HOMA-IR over time. Orchiectomy benefits more in SAT increases compared with LHRH agonists. Although no statistical significance was observed, the mean value of testosterone in orchiectomy group was higher than that in the LHRH agonist group. The progression of HOMA-IR was poorer among patients receiving LHRH agonists compared to the patients who underwent BO. This might be attributed to a greater reduction in adrenal androgens in the LHRH agonist group. A statistically significant reduction (p<0.01) in dehydroepiandrosterone level was observed in the group of medication treatment but not in the orchiectomy group. We may conclude that LHRH agonists cause a remarkable reduction in adrenal androgens, thus resulting in poorer glucose metabolism (14). These symptoms could be ameliorated, prevented, or even reversed by exercise and nutrition supplementation (37). Further feasible and effective strategy of specific exercise and nutrition supplementation should be investigated in future studies.

Therapeutic effects comparisons were mostly discussed in current studies, while metabolic disorder differences were only evaluated in three studies. This systematic review concluded several important gaps in current studies. Firstly, the choice of ADT approach was associated with various factors, and an assessment scale should be made for decision. Secondly, further studies directly comparing metabolic disorder between medication and surgical ADT are required. Thirdly, long-term follow-up studies should be performed among patients undergoing ADT to investigate related complications. Furthermore, studies on the treatment for adverse effects should be conducted in the future.

## 5 Conclusion

LHRH agonists and orchiectomy shared similar therapeutic effects for castration in PCa patients. Regarding adverse effects, both approaches had similar incidence of hot flash and lipid or glucose metabolic disorders. The metabolic disorder of LHRH agonists might be worse than that of the surgical approach, which might be attributed to the marked reduction of testosterone produced in the adrenal. Further studies directly comparing the long-term metabolic disorders should be performed. The therapeutic effects, adverse events, psychological and economical effects should be comprehensively considered when applying ADT methods.

## Data availability statement

The original contributions presented in the study are included in the article/**Supplementary Material**. Further inquiries can be directed to the corresponding author.

## Author contributions

XZ: Protocol/project development, Data management, Data analysis, Manuscript writing/editing; GZ: Data management, Data collection, Manuscript writing/editing; JW: Data collection, Manuscript writing/editing; YW: Protocol/project development, Data analysis, Manuscript writing/editing. All authors contributed to the article and approved the submitted version.

## References

[1] McLeod DG . Antiandrogenic drugs. Cancer (1993) 71(3 Suppl):1046–9. doi: 10.1002/1097-0142(19930201)71:3+<1046::AID-CNCR2820711424>3.0.CO;2-M 8428326

[2] el-Rayes BF Hussain MH . Hormonal therapy for prostate cancer: past, present and future. Expert Rev Anticancer Ther (2002) 2(1):37–47. doi: 10.1586/14737140.2.1.37 12113064

[3] Page MJ McKenzie JE Bossuyt PM Boutron I Hoffmann TC Mulrow CD . The PRISMA 2020 statement: an updated guideline for reporting systematic reviews. Bmj (2021) 372:n71. doi: 10.1136/bmj.n71 33782057 PMC8005924

[4] Luteinizing hormone-releasing hormone agonists versus orchiectomy in the treatment of prostate cancer: A systematic review and meta-analysis (2022). Available at: https://www.crd.york.ac.uk/PROSPERO/ (Accessed October 20, 2022). 10.3389/fendo.2023.1131715 PMC9939757 36814583

[5] Agarwala A Bansal S Gupta NP . Bilateral orchidectomy revisited in management of metastatic hormone-sensitive prostate cancer. Indian J Surg Oncol (2021) 12(3):565–70. doi: 10.1007/s13193-021-01390-w PMC8490498 34658587

[6] Kaisary AV Tyrrell CJ Peeling WB Griffiths K . Comparison of LHRH analogue (Zoladex) with orchiectomy in patients with metastatic prostatic carcinoma. Br J Urol (1991) 67(5):502–8. doi: 10.1111/j.1464-410X.1991.tb15195.x 1828183

[7] Østergren PB Kistorp C Fode M Henderson J Bennedbæk FN Faber J . Luteinizing hormone-releasing hormone agonists are superior to subcapsular orchiectomy in lowering testosterone levels of men with prostate cancer: Results from a randomized clinical trial. J Urol (2017) 197(6):1441–7. doi: 10.1016/j.juro.2016.12.003 PMC5433917 27939836

[8] Østergren PB Kistorp C Fode M Bennedbaek FN Faber J Sønksen J . Metabolic consequences of gonadotropin-releasing hormone agonists vs orchiectomy: a randomized clinical study. BJU Int (2019) 123(4):602–11. doi: 10.1111/bju.14609 30388320

[9] Parmar H Phillips RH Lightman SL Edwards L Allen L Schally AV . Randomised controlled study of orchidectomy vs long-acting d-Trp-6-LHRH microcapsules in advanced prostatic carcinoma. Lancet (1985) 2(8466):1201–5. doi: 10.1016/S0140-6736(85)90739-1 2866289

[10] Parmar H Edwards L Phillips RH Allen L Lightman SL . Orchiectomy versus long-acting d-Trp-6-LHRH in advanced prostatic cancer. Br J Urol (1987) 59(3):248–54. doi: 10.1136/bmj.n71 2952213

[11] Ryan PG Peeling WBUK . Trials of treatment for M1 prostatic cancer. the LH-RH analogue zoladex vs. orchidectomy. Am J Clin Oncol (1988) 11 Suppl 2:S169–72. doi: 10.1097/00000421-198801102-00039 2977272

[12] Selvi I Basar H . Subcapsular orchiectomy versus total orchiectomy and LHRH analogue in the treatment of hormone-sensitive metastatic prostate cancer: a different perspective in evaluation of the psychosocial effects. Support Care Cancer (2020) 28(9):4313–26. doi: 10.1007/s00520-019-05266-2 31912363

[13] Soloway MS Chodak G Vogelzang NJ Block NL Schellhammer PF Smith JA Jr. . Zoladex versus orchiectomy in treatment of advanced prostate cancer: a randomized trial. zoladex prostate study group. Urology (1991) 37(1):46–51. doi: 10.1016/0090-4295(91)80077-k 1824732

[14] Vargas A Machado RD Filho DI Paiva CE Dos Reis RB Tobias-Machado M . LHRH analog therapy is associated with worse metabolic side effects than bilateral orchiectomy in prostate cancer. World J Urol (2016) 34(12):1621–8. doi: 10.1007/s00345-016-1831-5 27103427

[15] Vogelzang NJ Chodak GW Soloway MS Block NL Schellhammer PF Smith JA Jr. . Goserelin versus orchiectomy in the treatment of advanced prostate cancer: final results of a randomized trial. zoladex prostate study group. Urology (1995) 46(2):220–6. doi: 10.1016/S0090-4295(99)80197-6 7624991

[16] Garje R Chennamadhavuni A Mott SL Chambers IM Gellhaus P Zakharia Y . Utilization and outcomes of surgical castration in comparison to medical castration in metastatic prostate cancer. Clin Genitourin Cancer (2020) 18(2):e157–e66. doi: 10.1016/j.clgc.2019.09.020 PMC7190190 31956009

[17] Huggins C Stevens RE Hodges CV . Studies on prostatic cancer: II. the effects of castration on advanced carcinoma of the prostate gland. Arch Surg (1941) 43(2): 209–223. doi: 10.1001/archsurg.1941.01210140043004

[18] Orakwe DE Tijani KH Jeje EA Ogunjimi MA Rufus WO Alabi TO . Bilateral subcapsular orchiectomy versus bilateral total orchiectomy: Comparison of the quality of life post-orchiectomy. Niger Postgrad Med J (2018) 25(1):43–7. doi: 10.4103/npmj.npmj_169_17 29676345

[19] Singh O Mukherjee P Sakthivel MS Wann C George AJP Gopalakrishnan R . Satisfaction and genital perception after orchiectomy for prostate cancer: does the technique matter? a randomized trial. Int Urol Nephrol (2021) 53(8):1583–9. doi: 10.1007/s11255-021-02849-z 33851360

[20] Issa MM Lendvay TS Bouet R Young MR Petros JA Marshall FF . Epididymal sparing bilateral simple orchiectomy with epididymoplasty: preservation of esthetics and body image. J Urol (2005) 174(3):893–7. doi: 10.1097/01.ju.0000172567.09442.b0 16093982

[21] Maatman TJ Gupta MK Montie JE . Effectiveness of castration versus intravenous estrogen therapy in producing rapid endocrine control of metastatic cancer of the prostate. J Urol (1985) 133(4):620–1. doi: 10.1016/S0022-5347(17)49115-4 3981712

[22] Atta MA Elabbady A Sameh W Sharafeldeen M Elsaqa M . Is there still a role for bilateral orchidectomy in androgen-deprivation therapy for metastatic prostate cancer? Arab J Urol (2020) 18(1):9–13. doi: 10.1080/2090598X.2019.1690270 32082628 PMC7006720

[23] Vetterlein MW Seisen T Löppenberg B Hanna N Cheng PJ Fisch M . Resident involvement in radical inguinal orchiectomy for testicular cancer does not adversely impact perioperative outcomes - a retrospective study. Urol Int (2017) 98(4):472–7. doi: 10.1159/000448596 27577733

[24] Cassileth BR Soloway MS Vogelzang NJ Schellhammer PS Seidmon EJ Hait HI . Patients' choice of treatment in stage d prostate cancer. Urology (1989) 33(5 Suppl):57–62. doi: 10.1016/0090-4295(89)90108-8 2523612

[25] Vihko P Härkönen P Soronen P Törn S Herrala A Kurkela R . 17 beta-hydroxysteroid dehydrogenases–their role in pathophysiology. Mol Cell Endocrinol (2004) 215(1-2):83–8. doi: 10.1016/j.mce.2003.11.021 15026178

[26] Robertson CN Roberson KM Padilla GM O'Brien ET Cook JM Kim CS . Induction of apoptosis by diethylstilbestrol in hormone-insensitive prostate cancer cells. J Natl Cancer Inst (1996) 88(13):908–17. doi: 10.1093/jnci/88.13.908 8656443

[27] Hill P Garbaczewski L Walker AR . Age, environmental factors and prostatic cancer. Med Hypotheses (1984) 14(1):29–39. doi: 10.1016/0306-9877(84)90060-4 6748989

[28] Kitahara S Umeda H Yano M Koga F Sumi S Moriguchi H . Effects of intravenous administration of high dose-diethylstilbestrol diphosphate on serum hormonal levels in patients with hormone-refractory prostate cancer. Endocr J (1999) 46(5):659–64. doi: 10.1507/endocrj.46.659 10670751

[29] Moreau JP Delavault P Blumberg J . Luteinizing hormone-releasing hormone agonists in the treatment of prostate cancer: a review of their discovery, development, and place in therapy. Clin Ther (2006) 28(10):1485–508. doi: 10.1016/j.clinthera.2006.10.018 17157109

[30] Higano CS . Side effects of androgen deprivation therapy: monitoring and minimizing toxicity. Urology (2003) 61(2 Suppl 1):32–8. doi: 10.1016/S0090-4295(02)02397-X 12667885

[31] Raina R Pahalajani G Agarwal A Zippe C . Long-term effectiveness of luteinizing hormone-releasing hormone agonist or antiandrogen monotherapy in elderly men with localized prostate cancer (T1-2): a retrospective study. Asian J Androl (2007) 9(2):253–8. doi: 10.1111/j.1745-7262.2007.00074.x 17334592

[32] Spetz AC Hammar M Lindberg B Spångberg A Varenhorst E . Prospective evaluation of hot flashes during treatment with parenteral estrogen or complete androgen ablation for metastatic carcinoma of the prostate. J Urol (2001) 166(2):517–20. doi: 10.1016/S0022-5347(05)65973-3 11458057

[33] Saylor PJ Smith MR . Metabolic complications of androgen deprivation therapy for prostate cancer. J Urol (2013) 189(1 Suppl):S34–42; discussion S3-4. doi: 10.1016/j.juro.2012.11.017 23234628

[34] Saad F Aversa A Isidori AM Gooren LJ . Testosterone as potential effective therapy in treatment of obesity in men with testosterone deficiency: A review. Curr Diabetes Rev (2012) 8(2):131–43. doi: 10.2174/157339912799424573 PMC3296126 22268394

[35] Oh JY Barrett-Connor E Wedick NM Wingard DL . Endogenous sex hormones and the development of type 2 diabetes in older men and women: the rancho Bernardo study. Diabetes Care (2002) 25(1):55–60. doi: 10.2337/diacare.25.1.55 11772901

[36] Galvão DA Spry NA Taaffe DR Newton RU Stanley J Shannon T . Changes in muscle, fat and bone mass after 36 weeks of maximal androgen blockade for prostate cancer. BJU Int (2008) 102(1):44–7. doi: 10.1111/j.1464-410X.2008.07539.x 18336606

[37] Wilson RL Taaffe DR Newton RU Hart NH Lyons-Wall P Galvão DA . Using exercise and nutrition to alter fat and lean mass in men with prostate cancer receiving androgen deprivation therapy: A narrative review. Nutrients (2021) 13(5):1664. doi: 10.3390/nu13051664 34068965 PMC8156712

