# Supplementary material for: Luteinizing hormone-releasing hormone agonists versus orchiectomy in the treatment of prostate cancer: A systematic review
Source: Front Endocrinol (Lausanne). 2023 Feb 6;14:1131715. doi: 10.3389/fendo.2023.1131715 (PMC9939757; doi:10.3389/fendo.2023.1131715)
Supplement: Supplementary file 1 [file Table_1.docx]

Supplementary Material

Luteinizing Hormone-Releasing Hormone Agonists versus Orchiectomy in the treatment of prostate cancer: A Systematic Review

Xianlu Zhang^1^, Gejun Zhang^1^, Jianfeng Wang^1^, Yanli Wang^2*^

*** Correspondence:** Yanli Wang, yanbao213@163.com

# Table S1 Search strategy

| Pubmed | | | | |
| --- | --- | --- | --- | --- |
| Search number | Query | Search details | Results | Time |
| 12 | #10 AND #11 | ("prostatic neoplasms"[MeSH Terms] OR ("prostate neoplasms"[Title/Abstract] OR "neoplasms prostate"[Title/Abstract] OR "neoplasm prostate"[Title/Abstract] OR "prostate neoplasm"[Title/Abstract] OR "neoplasms prostatic"[Title/Abstract] OR "neoplasm prostatic"[Title/Abstract] OR "prostatic neoplasm"[Title/Abstract] OR "prostate cancer"[Title/Abstract] OR "cancer prostate"[Title/Abstract] OR "cancers prostate"[Title/Abstract] OR "prostate cancers"[Title/Abstract] OR "cancer of the prostate"[Title/Abstract] OR "prostatic cancer"[Title/Abstract] OR "cancer prostatic"[Title/Abstract] OR "cancers prostatic"[Title/Abstract] OR "prostatic cancers"[Title/Abstract] OR "cancer of prostate"[Title/Abstract] OR "prostate cancer"[Title/Abstract] OR "cancer prostate"[Title/Abstract] OR "malignant prostate tumor"[Title/Abstract] OR "malignant prostate tumour"[Title/Abstract] OR "malignant prostatic tumor"[Title/Abstract] OR "malignant prostatic tumour"[Title/Abstract] OR "prostate gland cancer"[Title/Abstract] OR "prostate malignancy"[Title/Abstract] OR (("prostat"[All Fields] OR "prostate"[MeSH Terms] OR "prostate"[All Fields] OR "prostates"[All Fields] OR "prostatic"[All Fields] OR "prostatism"[MeSH Terms] OR "prostatism"[All Fields] OR "prostatitis"[MeSH Terms] OR "prostatitis"[All Fields]) AND "malignant neoplasm"[Title/Abstract]) OR "prostate malignant tumor"[Title/Abstract] OR (("prostat"[All Fields] OR "prostate"[MeSH Terms] OR "prostate"[All Fields] OR "prostates"[All Fields] OR "prostatic"[All Fields] OR "prostatism"[MeSH Terms] OR "prostatism"[All Fields] OR "prostatitis"[MeSH Terms] OR "prostatitis"[All Fields]) AND "malignant tumour"[Title/Abstract]) OR "prostatic cancer"[Title/Abstract] OR "prostatic malignancy"[Title/Abstract])) AND ("gonadotropin releasing hormone"[MeSH Terms] OR ("gonadotropin releasing hormone"[Title/Abstract] OR "gn rh"[Title/Abstract] OR "gonadoliberin"[Title/Abstract] OR ("lhfsh"[All Fields] AND "releasing hormone"[Title/Abstract]) OR ((("patient discharge"[MeSH Terms] OR ("patient"[All Fields] AND "discharge"[All Fields]) OR "patient discharge"[All Fields] OR "release"[All Fields] OR "released"[All Fields] OR "releases"[All Fields] OR "Releasing"[All Fields]) AND ("hormon"[All Fields] OR "hormonal"[All Fields] OR "hormonally"[All Fields] OR "hormonals"[All Fields] OR "hormone s"[All Fields] OR "hormones"[Pharmacological Action] OR "hormones"[MeSH Terms] OR "hormones"[All Fields] OR "Hormone"[All Fields] OR "hormons"[All Fields])) AND "lhfsh"[Title/Abstract]) OR "lh rh"[Title/Abstract] OR "lfrh"[Title/Abstract] OR "lh releasing hormone"[Title/Abstract] OR "lh releasing hormone"[Title/Abstract] OR "lh fsh releasing hormone"[Title/Abstract] OR "lh fsh releasing hormone"[Title/Abstract] OR "lhfshrh"[Title/Abstract] OR "lhrh"[Title/Abstract] OR "luliberin"[Title/Abstract] OR "gonadorelin"[Title/Abstract] OR "luteinizing hormone releasing hormone"[Title/Abstract] OR "luteinizing hormone releasing hormone"[Title/Abstract] OR "fsh releasing hormone"[Title/Abstract] OR "fsh releasing hormone"[Title/Abstract] OR "gnrh"[Title/Abstract] OR "factrel"[Title/Abstract] OR "cystorelin"[Title/Abstract] OR "gonadorelin hydrochloride"[Title/Abstract] OR "kryptocur"[Title/Abstract] OR "dirigestran"[Title/Abstract] OR "gonadorelin acetate"[Title/Abstract])) AND ("orchiectomy"[MeSH Terms] OR ("orchiectomies"[Title/Abstract] OR "orchidectomy"[Title/Abstract] OR "orchidectomies"[Title/Abstract] OR "castration male"[Title/Abstract] OR (("castrate"[All Fields] OR "castrated"[All Fields] OR "castrates"[All Fields] OR "castrating"[All Fields] OR "castration"[MeSH Terms] OR "castration"[All Fields] OR "castrations"[All Fields] OR "castrator"[All Fields] OR "castrators"[All Fields] OR "orchiectomy"[MeSH Terms] OR "orchiectomy"[All Fields]) AND "male"[Title/Abstract]) OR "male castration"[Title/Abstract] OR (("male"[MeSH Terms] OR "male"[All Fields]) AND "castrations"[Title/Abstract]) OR "orchectomy"[Title/Abstract] OR "orchidectomy"[Title/Abstract] OR "testectomy"[Title/Abstract])) AND "cohort"[Title/Abstract] | 64 | 2:15:06 |
| 11 | ('random*'[Title/Abstract]) OR ('cohort'[Title/Abstract]) | "cohort"[Title/Abstract] | 721,444 | 2:14:30 |
| 10 | #7 AND #8 AND #9 | ("prostatic neoplasms"[MeSH Terms] OR ("prostate neoplasms"[Title/Abstract] OR "neoplasms prostate"[Title/Abstract] OR "neoplasm prostate"[Title/Abstract] OR "prostate neoplasm"[Title/Abstract] OR "neoplasms prostatic"[Title/Abstract] OR "neoplasm prostatic"[Title/Abstract] OR "prostatic neoplasm"[Title/Abstract] OR "prostate cancer"[Title/Abstract] OR "cancer prostate"[Title/Abstract] OR "cancers prostate"[Title/Abstract] OR "prostate cancers"[Title/Abstract] OR "cancer of the prostate"[Title/Abstract] OR "prostatic cancer"[Title/Abstract] OR "cancer prostatic"[Title/Abstract] OR "cancers prostatic"[Title/Abstract] OR "prostatic cancers"[Title/Abstract] OR "cancer of prostate"[Title/Abstract] OR "prostate cancer"[Title/Abstract] OR "cancer prostate"[Title/Abstract] OR "malignant prostate tumor"[Title/Abstract] OR "malignant prostate tumour"[Title/Abstract] OR "malignant prostatic tumor"[Title/Abstract] OR "malignant prostatic tumour"[Title/Abstract] OR "prostate gland cancer"[Title/Abstract] OR "prostate malignancy"[Title/Abstract] OR (("prostat"[All Fields] OR "prostate"[MeSH Terms] OR "prostate"[All Fields] OR "prostates"[All Fields] OR "prostatic"[All Fields] OR "prostatism"[MeSH Terms] OR "prostatism"[All Fields] OR "prostatitis"[MeSH Terms] OR "prostatitis"[All Fields]) AND "malignant neoplasm"[Title/Abstract]) OR "prostate malignant tumor"[Title/Abstract] OR (("prostat"[All Fields] OR "prostate"[MeSH Terms] OR "prostate"[All Fields] OR "prostates"[All Fields] OR "prostatic"[All Fields] OR "prostatism"[MeSH Terms] OR "prostatism"[All Fields] OR "prostatitis"[MeSH Terms] OR "prostatitis"[All Fields]) AND "malignant tumour"[Title/Abstract]) OR "prostatic cancer"[Title/Abstract] OR "prostatic malignancy"[Title/Abstract])) AND ("gonadotropin releasing hormone"[MeSH Terms] OR ("gonadotropin releasing hormone"[Title/Abstract] OR "gn rh"[Title/Abstract] OR "gonadoliberin"[Title/Abstract] OR ("lhfsh"[All Fields] AND "releasing hormone"[Title/Abstract]) OR ((("patient discharge"[MeSH Terms] OR ("patient"[All Fields] AND "discharge"[All Fields]) OR "patient discharge"[All Fields] OR "release"[All Fields] OR "released"[All Fields] OR "releases"[All Fields] OR "Releasing"[All Fields]) AND ("hormon"[All Fields] OR "hormonal"[All Fields] OR "hormonally"[All Fields] OR "hormonals"[All Fields] OR "hormone s"[All Fields] OR "hormones"[Pharmacological Action] OR "hormones"[MeSH Terms] OR "hormones"[All Fields] OR "Hormone"[All Fields] OR "hormons"[All Fields])) AND "lhfsh"[Title/Abstract]) OR "lh rh"[Title/Abstract] OR "lfrh"[Title/Abstract] OR "lh releasing hormone"[Title/Abstract] OR "lh releasing hormone"[Title/Abstract] OR "lh fsh releasing hormone"[Title/Abstract] OR "lh fsh releasing hormone"[Title/Abstract] OR "lhfshrh"[Title/Abstract] OR "lhrh"[Title/Abstract] OR "luliberin"[Title/Abstract] OR "gonadorelin"[Title/Abstract] OR "luteinizing hormone releasing hormone"[Title/Abstract] OR "luteinizing hormone releasing hormone"[Title/Abstract] OR "fsh releasing hormone"[Title/Abstract] OR "fsh releasing hormone"[Title/Abstract] OR "gnrh"[Title/Abstract] OR "factrel"[Title/Abstract] OR "cystorelin"[Title/Abstract] OR "gonadorelin hydrochloride"[Title/Abstract] OR "kryptocur"[Title/Abstract] OR "dirigestran"[Title/Abstract] OR "gonadorelin acetate"[Title/Abstract])) AND ("orchiectomy"[MeSH Terms] OR ("orchiectomies"[Title/Abstract] OR "orchidectomy"[Title/Abstract] OR "orchidectomies"[Title/Abstract] OR "castration male"[Title/Abstract] OR (("castrate"[All Fields] OR "castrated"[All Fields] OR "castrates"[All Fields] OR "castrating"[All Fields] OR "castration"[MeSH Terms] OR "castration"[All Fields] OR "castrations"[All Fields] OR "castrator"[All Fields] OR "castrators"[All Fields] OR "orchiectomy"[MeSH Terms] OR "orchiectomy"[All Fields]) AND "male"[Title/Abstract]) OR "male castration"[Title/Abstract] OR (("male"[MeSH Terms] OR "male"[All Fields]) AND "castrations"[Title/Abstract]) OR "orchectomy"[Title/Abstract] OR "orchidectomy"[Title/Abstract] OR "testectomy"[Title/Abstract])) | 891 | 2:13:37 |
| 9 | #5 OR #6 | "orchiectomy"[MeSH Terms] OR ("orchiectomies"[Title/Abstract] OR "orchidectomy"[Title/Abstract] OR "orchidectomies"[Title/Abstract] OR "castration male"[Title/Abstract] OR (("castrate"[All Fields] OR "castrated"[All Fields] OR "castrates"[All Fields] OR "castrating"[All Fields] OR "castration"[MeSH Terms] OR "castration"[All Fields] OR "castrations"[All Fields] OR "castrator"[All Fields] OR "castrators"[All Fields] OR "orchiectomy"[MeSH Terms] OR "orchiectomy"[All Fields]) AND "male"[Title/Abstract]) OR "male castration"[Title/Abstract] OR (("male"[MeSH Terms] OR "male"[All Fields]) AND "castrations"[Title/Abstract]) OR "orchectomy"[Title/Abstract] OR "orchidectomy"[Title/Abstract] OR "testectomy"[Title/Abstract]) | 27,693 | 2:13:13 |
| 8 | #3 OR #4 | "gonadotropin releasing hormone"[MeSH Terms] OR ("gonadotropin releasing hormone"[Title/Abstract] OR "gn rh"[Title/Abstract] OR "gonadoliberin"[Title/Abstract] OR ("lhfsh"[All Fields] AND "releasing hormone"[Title/Abstract]) OR ((("patient discharge"[MeSH Terms] OR ("patient"[All Fields] AND "discharge"[All Fields]) OR "patient discharge"[All Fields] OR "release"[All Fields] OR "released"[All Fields] OR "releases"[All Fields] OR "Releasing"[All Fields]) AND ("hormon"[All Fields] OR "hormonal"[All Fields] OR "hormonally"[All Fields] OR "hormonals"[All Fields] OR "hormone s"[All Fields] OR "hormones"[Pharmacological Action] OR "hormones"[MeSH Terms] OR "hormones"[All Fields] OR "Hormone"[All Fields] OR "hormons"[All Fields])) AND "lhfsh"[Title/Abstract]) OR "lh rh"[Title/Abstract] OR "lfrh"[Title/Abstract] OR "lh releasing hormone"[Title/Abstract] OR "lh releasing hormone"[Title/Abstract] OR "lh fsh releasing hormone"[Title/Abstract] OR "lh fsh releasing hormone"[Title/Abstract] OR "lhfshrh"[Title/Abstract] OR "lhrh"[Title/Abstract] OR "luliberin"[Title/Abstract] OR "gonadorelin"[Title/Abstract] OR "luteinizing hormone releasing hormone"[Title/Abstract] OR "luteinizing hormone releasing hormone"[Title/Abstract] OR "fsh releasing hormone"[Title/Abstract] OR "fsh releasing hormone"[Title/Abstract] OR "gnrh"[Title/Abstract] OR "factrel"[Title/Abstract] OR "cystorelin"[Title/Abstract] OR "gonadorelin hydrochloride"[Title/Abstract] OR "kryptocur"[Title/Abstract] OR "dirigestran"[Title/Abstract] OR "gonadorelin acetate"[Title/Abstract]) | 48,446 | 2:13:05 |
| 7 | #1 OR #2 | "prostatic neoplasms"[MeSH Terms] OR ("prostate neoplasms"[Title/Abstract] OR "neoplasms prostate"[Title/Abstract] OR "neoplasm prostate"[Title/Abstract] OR "prostate neoplasm"[Title/Abstract] OR "neoplasms prostatic"[Title/Abstract] OR "neoplasm prostatic"[Title/Abstract] OR "prostatic neoplasm"[Title/Abstract] OR "prostate cancer"[Title/Abstract] OR "cancer prostate"[Title/Abstract] OR "cancers prostate"[Title/Abstract] OR "prostate cancers"[Title/Abstract] OR "cancer of the prostate"[Title/Abstract] OR "prostatic cancer"[Title/Abstract] OR "cancer prostatic"[Title/Abstract] OR "cancers prostatic"[Title/Abstract] OR "prostatic cancers"[Title/Abstract] OR "cancer of prostate"[Title/Abstract] OR "prostate cancer"[Title/Abstract] OR "cancer prostate"[Title/Abstract] OR "malignant prostate tumor"[Title/Abstract] OR "malignant prostate tumour"[Title/Abstract] OR "malignant prostatic tumor"[Title/Abstract] OR "malignant prostatic tumour"[Title/Abstract] OR "prostate gland cancer"[Title/Abstract] OR "prostate malignancy"[Title/Abstract] OR (("prostat"[All Fields] OR "prostate"[MeSH Terms] OR "prostate"[All Fields] OR "prostates"[All Fields] OR "prostatic"[All Fields] OR "prostatism"[MeSH Terms] OR "prostatism"[All Fields] OR "prostatitis"[MeSH Terms] OR "prostatitis"[All Fields]) AND "malignant neoplasm"[Title/Abstract]) OR "prostate malignant tumor"[Title/Abstract] OR (("prostat"[All Fields] OR "prostate"[MeSH Terms] OR "prostate"[All Fields] OR "prostates"[All Fields] OR "prostatic"[All Fields] OR "prostatism"[MeSH Terms] OR "prostatism"[All Fields] OR "prostatitis"[MeSH Terms] OR "prostatitis"[All Fields]) AND "malignant tumour"[Title/Abstract]) OR "prostatic cancer"[Title/Abstract] OR "prostatic malignancy"[Title/Abstract]) | 181,087 | 2:12:57 |
| 6 | ‘Orchiectomies’[Title/Abstract] OR ‘Orchidectomy’[Title/Abstract] OR ‘Orchidectomies’[Title/Abstract] OR ‘Castration, Male’[Title/Abstract] OR ‘Castrations, Male’[Title/Abstract] OR ‘Male Castration’[Title/Abstract] OR ‘Male Castrations’[Title/Abstract] OR ‘orchectomy’[Title/Abstract] OR ‘orcheotomy’[Title/Abstract] OR ‘orchidectomy’[Title/Abstract] OR ‘testectomy’[Title/Abstract] | "orchiectomies"[Title/Abstract] OR "orchidectomy"[Title/Abstract] OR "orchidectomies"[Title/Abstract] OR "castration male"[Title/Abstract] OR (("castrate"[All Fields] OR "castrated"[All Fields] OR "castrates"[All Fields] OR "castrating"[All Fields] OR "castration"[MeSH Terms] OR "castration"[All Fields] OR "castrations"[All Fields] OR "castrator"[All Fields] OR "castrators"[All Fields] OR "orchiectomy"[MeSH Terms] OR "orchiectomy"[All Fields]) AND "male"[Title/Abstract]) OR "male castration"[Title/Abstract] OR (("male"[MeSH Terms] OR "male"[All Fields]) AND "castrations"[Title/Abstract]) OR "orchectomy"[Title/Abstract] OR "orchidectomy"[Title/Abstract] OR "testectomy"[Title/Abstract] | 18,194 | 2:12:05 |
| 5 | Orchiectomy[MeSH Terms] | "orchiectomy"[MeSH Terms] | 15,385 | 2:11:48 |
| 4 | ‘Gonadotropin Releasing Hormone’[Title/Abstract] OR ‘Gn-RH’[Title/Abstract] OR ‘Gonadoliberin’[Title/Abstract] OR ‘LHFSH Releasing Hormone’[Title/Abstract] OR ‘Releasing Hormone, LHFSH’[Title/Abstract] OR ‘LH-RH’[Title/Abstract] OR ‘LFRH’[Title/Abstract] OR ‘LH-Releasing Hormone’[Title/Abstract] OR ‘LH Releasing Hormone’[Title/Abstract] OR ‘LH-FSH Releasing Hormone’[Title/Abstract] OR ‘LH FSH Releasing Hormone’[Title/Abstract] OR ‘LHFSHRH’[Title/Abstract] OR ‘LHRH’[Title/Abstract] OR ‘Luliberin’[Title/Abstract] OR ‘Gonadorelin’[Title/Abstract] OR ‘Luteinizing Hormone-Releasing Hormone’[Title/Abstract] OR ‘Luteinizing Hormone Releasing Hormone’[Title/Abstract] OR ‘FSH-Releasing Hormone’[Title/Abstract] OR ‘FSH Releasing Hormone’[Title/Abstract] OR ‘GnRH’[Title/Abstract] OR ‘Factrel’[Title/Abstract] OR ‘Cystorelin’[Title/Abstract] OR ‘Gonadorelin Hydrochloride’[Title/Abstract] OR ‘Kryptocur’[Title/Abstract] OR ‘Dirigestran’[Title/Abstract] OR ‘Gonadorelin Acetate’[Title/Abstract] | "gonadotropin releasing hormone"[Title/Abstract] OR "gn rh"[Title/Abstract] OR "gonadoliberin"[Title/Abstract] OR ("lhfsh"[All Fields] AND "releasing hormone"[Title/Abstract]) OR ((("patient discharge"[MeSH Terms] OR ("patient"[All Fields] AND "discharge"[All Fields]) OR "patient discharge"[All Fields] OR "release"[All Fields] OR "released"[All Fields] OR "releases"[All Fields] OR "Releasing"[All Fields]) AND ("hormon"[All Fields] OR "hormonal"[All Fields] OR "hormonally"[All Fields] OR "hormonals"[All Fields] OR "hormone s"[All Fields] OR "hormones"[Pharmacological Action] OR "hormones"[MeSH Terms] OR "hormones"[All Fields] OR "Hormone"[All Fields] OR "hormons"[All Fields])) AND "lhfsh"[Title/Abstract]) OR "lh rh"[Title/Abstract] OR "lfrh"[Title/Abstract] OR "lh releasing hormone"[Title/Abstract] OR "lh releasing hormone"[Title/Abstract] OR "lh fsh releasing hormone"[Title/Abstract] OR "lh fsh releasing hormone"[Title/Abstract] OR "lhfshrh"[Title/Abstract] OR "lhrh"[Title/Abstract] OR "luliberin"[Title/Abstract] OR "gonadorelin"[Title/Abstract] OR "luteinizing hormone releasing hormone"[Title/Abstract] OR "luteinizing hormone releasing hormone"[Title/Abstract] OR "fsh releasing hormone"[Title/Abstract] OR "fsh releasing hormone"[Title/Abstract] OR "gnrh"[Title/Abstract] OR "factrel"[Title/Abstract] OR "cystorelin"[Title/Abstract] OR "gonadorelin hydrochloride"[Title/Abstract] OR "kryptocur"[Title/Abstract] OR "dirigestran"[Title/Abstract] OR "gonadorelin acetate"[Title/Abstract] | 40,192 | 2:11:26 |
| 3 | Gonadotropin-Releasing Hormone[MeSH Terms] | "gonadotropin releasing hormone"[MeSH Terms] | 33,895 | 2:08:57 |
| 2 | ‘Prostate Neoplasms’[Title/Abstract] OR ‘Neoplasms, Prostate’[Title/Abstract] OR ‘Neoplasm, Prostate’[Title/Abstract] OR ‘Prostate Neoplasm’[Title/Abstract] OR ‘Neoplasms, Prostatic’[Title/Abstract] OR ‘Neoplasm, Prostatic’[Title/Abstract] OR ‘Prostatic Neoplasm’[Title/Abstract] OR ‘Prostate Cancer’[Title/Abstract] OR ‘Cancer, Prostate’[Title/Abstract] OR ‘Cancers, Prostate’[Title/Abstract] OR ‘Prostate Cancers’[Title/Abstract] OR ‘Cancer of the Prostate’[Title/Abstract] OR ‘Prostatic Cancer’[Title/Abstract] OR ‘Cancer, Prostatic’[Title/Abstract] OR ‘Cancers, Prostatic’[Title/Abstract] OR ‘Prostatic Cancers’[Title/Abstract] OR ‘Cancer of Prostate’[Title/Abstract] OR ‘prostate cancer’[Title/Abstract] OR ‘cancer prostate’[Title/Abstract] OR ‘malignant prostate tumor’[Title/Abstract] OR ‘malignant prostate tumour’[Title/Abstract] OR ‘malignant prostatic tumor’[Title/Abstract] OR ‘malignant prostatic tumour’[Title/Abstract] OR ‘prostate gland cancer’[Title/Abstract] OR ‘prostate malignancy’[Title/Abstract] OR ‘prostate malignant neoplasm’[Title/Abstract] OR ‘prostate malignant tumor’[Title/Abstract] OR ‘prostate malignant tumour’[Title/Abstract] OR ‘prostatic cancer’[Title/Abstract] OR ‘prostatic malignancy’[Title/Abstract] | "prostate neoplasms"[Title/Abstract] OR "neoplasms prostate"[Title/Abstract] OR "neoplasm prostate"[Title/Abstract] OR "prostate neoplasm"[Title/Abstract] OR "neoplasms prostatic"[Title/Abstract] OR "neoplasm prostatic"[Title/Abstract] OR "prostatic neoplasm"[Title/Abstract] OR "prostate cancer"[Title/Abstract] OR "cancer prostate"[Title/Abstract] OR "cancers prostate"[Title/Abstract] OR "prostate cancers"[Title/Abstract] OR "cancer of the prostate"[Title/Abstract] OR "prostatic cancer"[Title/Abstract] OR "cancer prostatic"[Title/Abstract] OR "cancers prostatic"[Title/Abstract] OR "prostatic cancers"[Title/Abstract] OR "cancer of prostate"[Title/Abstract] OR "prostate cancer"[Title/Abstract] OR "cancer prostate"[Title/Abstract] OR "malignant prostate tumor"[Title/Abstract] OR "malignant prostate tumour"[Title/Abstract] OR "malignant prostatic tumor"[Title/Abstract] OR "malignant prostatic tumour"[Title/Abstract] OR "prostate gland cancer"[Title/Abstract] OR "prostate malignancy"[Title/Abstract] OR (("prostat"[All Fields] OR "prostate"[MeSH Terms] OR "prostate"[All Fields] OR "prostates"[All Fields] OR "prostatic"[All Fields] OR "prostatism"[MeSH Terms] OR "prostatism"[All Fields] OR "prostatitis"[MeSH Terms] OR "prostatitis"[All Fields]) AND "malignant neoplasm"[Title/Abstract]) OR "prostate malignant tumor"[Title/Abstract] OR (("prostat"[All Fields] OR "prostate"[MeSH Terms] OR "prostate"[All Fields] OR "prostates"[All Fields] OR "prostatic"[All Fields] OR "prostatism"[MeSH Terms] OR "prostatism"[All Fields] OR "prostatitis"[MeSH Terms] OR "prostatitis"[All Fields]) AND "malignant tumour"[Title/Abstract]) OR "prostatic cancer"[Title/Abstract] OR "prostatic malignancy"[Title/Abstract] | 145,088 | 2:08:35 |
| 1 | Prostatic Neoplasms[MeSH Terms] | "prostatic neoplasms"[MeSH Terms] | 144,392 | 2:08:10 |

| Embase |  |  |  |
| --- | --- | --- | --- |
| No. | Query | Results | Date |
| #12 | #10 AND #11 | 224 | 9-Oct-22 |
| #11 | 'random*':ab,ti,kw OR 'cohort':ab,ti,kw | 2962354 | 9-Oct-22 |
| #10 | #7 AND #8 AND #9 | 909 | 9-Oct-22 |
| #9 | #5 OR #6 | 20969 | 9-Oct-22 |
| #8 | #3 OR #4 | 67467 | 9-Oct-22 |
| #7 | #1 OR #2 | 292937 | 9-Oct-22 |
| #6 | 'orchiectomies':ab,ti,kw OR 'orchidectomies':ab,ti,kw OR 'castration, male':ab,ti,kw OR 'castrations, male':ab,ti,kw OR 'male castration':ab,ti,kw OR 'male castrations':ab,ti,kw OR 'orchectomy':ab,ti,kw OR 'orcheotomy':ab,ti,kw OR 'orchidectomy':ab,ti,kw OR 'testectomy':ab,ti,kw | 4178 | 9-Oct-22 |
| #5 | 'orchiectomy'/exp | 19913 | 9-Oct-22 |
| #4 | 'gn-rh':ab,ti,kw OR 'lhfsh releasing hormone':ab,ti,kw OR 'releasing hormone, lhfsh':ab,ti,kw OR 'lh-rh':ab,ti,kw OR 'lfrh':ab,ti,kw OR 'lh-releasing hormone':ab,ti,kw OR 'lh-fsh releasing hormone':ab,ti,kw OR 'lh fsh releasing hormone':ab,ti,kw OR 'lhfshrh':ab,ti,kw OR 'gonadorelin':ab,ti,kw OR 'luteinizing hormone-releasing hormone':ab,ti,kw OR 'fsh-releasing hormone':ab,ti,kw OR 'fsh releasing hormone':ab,ti,kw OR 'cystorelin':ab,ti,kw OR 'dirigestran':ab,ti,kw OR 'gonadorelin acetate':ab,ti,kw OR '6 leucine 10 deglycine gonadotropin releasing factor ethylamide':ab,ti,kw OR '[6 leucine 10 deglycine] gonadotropin releasing factor ethylamide':ab,ti,kw OR 'ay 24, 031':ab,ti,kw OR 'ay 24031':ab,ti,kw OR 'ay24, 031':ab,ti,kw OR 'ay24031':ab,ti,kw OR 'cryptocur':ab,ti,kw OR 'factrel':ab,ti,kw OR 'fertagyl':ab,ti,kw OR 'fertiral':ab,ti,kw OR 'gn rh':ab,ti,kw OR 'gnrh':ab,ti,kw OR 'gnrh serono':ab,ti,kw OR 'gonadoliberin':ab,ti,kw OR 'gonadorelin hydrochloride':ab,ti,kw OR 'gonadotrophin releasing factor':ab,ti,kw OR 'gonadotrophin releasing hormone':ab,ti,kw OR 'gonadotropin release factor':ab,ti,kw OR 'gonadotropin releasing factor':ab,ti,kw OR 'gonadotropin releasing hormone':ab,ti,kw OR 'gonadotropin-releasing hormone':ab,ti,kw OR 'hoe 471':ab,ti,kw OR 'hoe471':ab,ti,kw OR 'hoechst 471':ab,ti,kw OR 'ici 88262':ab,ti,kw OR 'ici88262':ab,ti,kw OR 'kryptocur':ab,ti,kw OR 'lh releasing hormone':ab,ti,kw OR 'lhrf':ab,ti,kw OR 'lhrh':ab,ti,kw OR 'lrh':ab,ti,kw OR 'lrh luteinising hormone releasing factor':ab,ti,kw OR 'lrh luteinizing hormone releasing factor':ab,ti,kw OR 'luforan':ab,ti,kw OR 'luliberin':ab,ti,kw OR 'luliberine':ab,ti,kw OR 'lutal':ab,ti,kw OR 'lutamin':ab,ti,kw OR 'luteinising hormone release factor':ab,ti,kw OR 'luteinising hormone releasing factor':ab,ti,kw OR 'luteinising hormone releasing hormone':ab,ti,kw OR 'luteinizing hormone release factor':ab,ti,kw OR 'luteinizing hormone releasing factor':ab,ti,kw OR 'luteinizing hormone releasing hormone':ab,ti,kw OR 'pulstim':ab,ti,kw OR 'relisorm':ab,ti,kw | 55945 | 9-Oct-22 |
| #3 | 'gonadorelin'/exp | 40301 | 9-Oct-22 |
| #2 | 'prostate neoplasms':ab,ti,kw OR 'neoplasms, prostate':ab,ti,kw OR 'neoplasm, prostate':ab,ti,kw OR 'prostate neoplasm':ab,ti,kw OR 'neoplasms, prostatic':ab,ti,kw OR 'neoplasm, prostatic':ab,ti,kw OR 'prostatic neoplasm':ab,ti,kw OR 'cancer, prostate':ab,ti,kw OR 'cancers, prostate':ab,ti,kw OR 'prostate cancers':ab,ti,kw OR 'cancer of the prostate':ab,ti,kw OR 'cancer, prostatic':ab,ti,kw OR 'cancers, prostatic':ab,ti,kw OR 'prostatic cancers':ab,ti,kw OR 'cancer of prostate':ab,ti,kw OR 'prostate cancer':ab,ti,kw OR 'cancer prostate':ab,ti,kw OR 'malignant prostate tumor':ab,ti,kw OR 'malignant prostate tumour':ab,ti,kw OR 'malignant prostatic tumor':ab,ti,kw OR 'malignant prostatic tumour':ab,ti,kw OR 'prostate gland cancer':ab,ti,kw OR 'prostate malignancy':ab,ti,kw OR 'prostate malignant neoplasm':ab,ti,kw OR 'prostate malignant tumor':ab,ti,kw OR 'prostate malignant tumour':ab,ti,kw OR 'prostatic cancer':ab,ti,kw OR 'prostatic malignancy':ab,ti,kw | 220013 | 9-Oct-22 |
| #1 | 'prostate cancer'/exp OR 'prostate cancer' | 288414 | 9-Oct-22 |

| Cochrane Library | | |
| --- | --- | --- |
| Date Run: 17/10/2022 04:32:27 | | |
| ID | Search | Hits |
| #1 | MeSH descriptor: [Prostatic Neoplasms] explode all trees | 6216 |
| #2 | (‘Prostate Neoplasms’ OR ‘Neoplasms, Prostate’ OR ‘Neoplasm, Prostate’ OR ‘Prostate Neoplasm’ OR ‘Neoplasms, Prostatic’ OR ‘Neoplasm, Prostatic’ OR ‘Prostatic Neoplasm’ OR ‘Prostate Cancer’ OR ‘Cancer, Prostate’ OR ‘Cancers, Prostate’ OR ‘Prostate Cancers’ OR ‘Cancer of the Prostate’ OR ‘Prostatic Cancer’ OR ‘Cancer, Prostatic’ OR ‘Cancers, Prostatic’ OR ‘Prostatic Cancers’ OR ‘Cancer of Prostate’ OR ‘prostate cancer’ OR ‘cancer prostate’ OR ‘malignant prostate tumor’ OR ‘malignant prostate tumour’ OR ‘malignant prostatic tumor’ OR ‘malignant prostatic tumour’ OR ‘prostate gland cancer’ OR ‘prostate malignancy’ OR ‘prostate malignant neoplasm’ OR ‘prostate malignant tumor’ OR ‘prostate malignant tumour’ OR ‘prostatic cancer’ OR ‘prostatic malignancy’):ti,ab,kw | 16437 |
| #3 | MeSH descriptor: [Gonadotropin-Releasing Hormone] explode all trees | 2772 |
| #4 | (‘Gonadotropin Releasing Hormone’ OR ‘Gn-RH’ OR ‘Gonadoliberin’ OR ‘LHFSH Releasing Hormone’ OR ‘Releasing Hormone, LHFSH’ OR ‘LH-RH’ OR ‘LFRH’ OR ‘LH-Releasing Hormone’ OR ‘LH Releasing Hormone’ OR ‘LH-FSH Releasing Hormone’ OR ‘LH FSH Releasing Hormone’ OR ‘LHFSHRH’ OR ‘LHRH’ OR ‘Luliberin’ OR ‘Gonadorelin’ OR ‘Luteinizing Hormone-Releasing Hormone’ OR ‘Luteinizing Hormone Releasing Hormone’ OR ‘FSH-Releasing Hormone’ OR ‘FSH Releasing Hormone’ OR ‘GnRH’ OR ‘Factrel’ OR ‘Cystorelin’ OR ‘Gonadorelin Hydrochloride’ OR ‘Kryptocur’ OR ‘Dirigestran’ OR ‘Gonadorelin Acetate’):ti,ab,kw | 6327 |
| #5 | MeSH descriptor: [Orchiectomy] explode all trees | 347 |
| #6 | (‘Orchiectomies’ OR ‘Orchidectomy’ OR ‘Orchidectomies’ OR ‘Castration, Male’ OR ‘Castrations, Male’ OR ‘Male Castration’ OR ‘Male Castrations’ OR ‘orchectomy’ OR ‘orcheotomy’ OR ‘orchidectomy’ OR ‘testectomy’):ti,ab,kw | 2217 |
| #7 | #1 OR #2 | 16437 |
| #8 | #3 OR #4 | 7029 |
| #9 | #5 OR #6 | 2385 |
| #10 | #7 AND #8 AND #9 | 340 |
| #11 | ('random*' OR 'cohort'):ti,ab,kw | 1188453 |
| #12 | #10 AND #11 | 284 |

| Web of Science | | |
| --- | --- | --- |
| ID | Search | Hits |
| #1 | TI=((Prostate Neoplasms) OR (Neoplasms, Prostate) OR (Neoplasm, Prostate) OR (Prostate Neoplasm) OR (Neoplasms, Prostatic) OR (Neoplasm, Prostatic) OR (Prostatic Neoplasm) OR (Prostate Cancer) OR (Cancer, Prostate) OR (Cancers, Prostate) OR (Prostate Cancers) OR (Cancer of the Prostate) OR (Prostatic Cancer) OR (Cancer, Prostatic) OR (Cancers, Prostatic) OR (Prostatic Cancers) OR (Cancer of Prostate) OR (prostate cancer) OR (cancer prostate) OR (malignant prostate tumor) OR (malignant prostate tumour) OR (malignant prostatic tumor) OR (malignant prostatic tumour) OR (prostate gland cancer) OR (prostate malignancy) OR (prostate malignant neoplasm) OR (prostate malignant tumor) OR (prostate malignant tumour) OR (prostatic cancer) OR (prostatic malignancy)) OR AK=((Prostate Neoplasms) OR (Neoplasms, Prostate) OR (Neoplasm, Prostate) OR (Prostate Neoplasm) OR (Neoplasms, Prostatic) OR (Neoplasm, Prostatic) OR (Prostatic Neoplasm) OR (Prostate Cancer) OR (Cancer, Prostate) OR (Cancers, Prostate) OR (Prostate Cancers) OR (Cancer of the Prostate) OR (Prostatic Cancer) OR (Cancer, Prostatic) OR (Cancers, Prostatic) OR (Prostatic Cancers) OR (Cancer of Prostate) OR (prostate cancer) OR (cancer prostate) OR (malignant prostate tumor) OR (malignant prostate tumour) OR (malignant prostatic tumor) OR (malignant prostatic tumour) OR (prostate gland cancer) OR (prostate malignancy) OR (prostate malignant neoplasm) OR (prostate malignant tumor) OR (prostate malignant tumour) OR (prostatic cancer) OR (prostatic malignancy)) OR AB=((Prostate Neoplasms) OR (Neoplasms, Prostate) OR (Neoplasm, Prostate) OR (Prostate Neoplasm) OR (Neoplasms, Prostatic) OR (Neoplasm, Prostatic) OR (Prostatic Neoplasm) OR (Prostate Cancer) OR (Cancer, Prostate) OR (Cancers, Prostate) OR (Prostate Cancers) OR (Cancer of the Prostate) OR (Prostatic Cancer) OR (Cancer, Prostatic) OR (Cancers, Prostatic) OR (Prostatic Cancers) OR (Cancer of Prostate) OR (prostate cancer) OR (cancer prostate) OR (malignant prostate tumor) OR (malignant prostate tumour) OR (malignant prostatic tumor) OR (malignant prostatic tumour) OR (prostate gland cancer) OR (prostate malignancy) OR (prostate malignant neoplasm) OR (prostate malignant tumor) OR (prostate malignant tumour) OR (prostatic cancer) OR (prostatic malignancy)) | 201,659 |
| #2 | TI=((Gonadotropin Releasing Hormone) OR (Gn-RH) OR (Gonadoliberin) OR (LHFSH Releasing Hormone) OR (Releasing Hormone, LHFSH) OR (LH-RH) OR (LFRH) OR (LH-Releasing Hormone) OR (LH Releasing Hormone) OR (LH-FSH Releasing Hormone) OR (LH FSH Releasing Hormone) OR (LHFSHRH) OR (LHRH) OR (Luliberin) OR (Gonadorelin) OR (Luteinizing Hormone-Releasing Hormone) OR (Luteinizing Hormone Releasing Hormone) OR (FSH-Releasing Hormone) OR (FSH Releasing Hormone) OR (GnRH) OR (Factrel) OR (Cystorelin) OR (Gonadorelin Hydrochloride) OR (Kryptocur) OR (Dirigestran) OR (Gonadorelin Acetate) OR (6 leucine 10 deglycine gonadotropin releasing factor ethylamide) OR ([6 leucine 10 deglycine] gonadotropin releasing factor ethylamide) OR (ay 24, 031) OR (ay 24031) OR (ay24, 031) OR (ay24031) OR (cryptocur) OR (factrel) OR (fertagyl) OR (fertiral) OR (gn rh) OR (gnrh) OR (gnrh serono) OR (gonadoliberin) OR (gonadorelin hydrochloride) OR (gonadotrophin releasing factor) OR (gonadotrophin releasing hormone) OR (gonadotropin release factor) OR (gonadotropin releasing factor) OR (gonadotropin releasing hormone) OR (gonadotropin-releasing hormone) OR (hoe 471) OR (hoe471) OR (hoechst 471) OR (ici 88262) OR (ici88262) OR (kryptocur) OR (lh releasing hormone) OR (lhrf) OR (LHRH) OR (lrh) OR (lrh luteinising hormone releasing factor) OR (lrh luteinizing hormone releasing factor) OR (luforan) OR (luliberin) OR (luliberine) OR (lutal) OR (lutamin) OR (luteinising hormone release factor) OR (luteinising hormone releasing factor) OR (luteinising hormone releasing hormone) OR (luteinizing hormone release factor) OR (luteinizing hormone releasing factor) OR (luteinizing hormone releasing hormone) OR (pulstim) OR (relisorm)) OR AK=((Gonadotropin Releasing Hormone) OR (Gn-RH) OR (Gonadoliberin) OR (LHFSH Releasing Hormone) OR (Releasing Hormone, LHFSH) OR (LH-RH) OR (LFRH) OR (LH-Releasing Hormone) OR (LH Releasing Hormone) OR (LH-FSH Releasing Hormone) OR (LH FSH Releasing Hormone) OR (LHFSHRH) OR (LHRH) OR (Luliberin) OR (Gonadorelin) OR (Luteinizing Hormone-Releasing Hormone) OR (Luteinizing Hormone Releasing Hormone) OR (FSH-Releasing Hormone) OR (FSH Releasing Hormone) OR (GnRH) OR (Factrel) OR (Cystorelin) OR (Gonadorelin Hydrochloride) OR (Kryptocur) OR (Dirigestran) OR (Gonadorelin Acetate) OR (6 leucine 10 deglycine gonadotropin releasing factor ethylamide) OR ([6 leucine 10 deglycine] gonadotropin releasing factor ethylamide) OR (ay 24, 031) OR (ay 24031) OR (ay24, 031) OR (ay24031) OR (cryptocur) OR (factrel) OR (fertagyl) OR (fertiral) OR (gn rh) OR (gnrh) OR (gnrh serono) OR (gonadoliberin) OR (gonadorelin hydrochloride) OR (gonadotrophin releasing factor) OR (gonadotrophin releasing hormone) OR (gonadotropin release factor) OR (gonadotropin releasing factor) OR (gonadotropin releasing hormone) OR (gonadotropin-releasing hormone) OR (hoe 471) OR (hoe471) OR (hoechst 471) OR (ici 88262) OR (ici88262) OR (kryptocur) OR (lh releasing hormone) OR (lhrf) OR (LHRH) OR (lrh) OR (lrh luteinising hormone releasing factor) OR (lrh luteinizing hormone releasing factor) OR (luforan) OR (luliberin) OR (luliberine) OR (lutal) OR (lutamin) OR (luteinising hormone release factor) OR (luteinising hormone releasing factor) OR (luteinising hormone releasing hormone) OR (luteinizing hormone release factor) OR (luteinizing hormone releasing factor) OR (luteinizing hormone releasing hormone) OR (pulstim) OR (relisorm)) OR AB=((Gonadotropin Releasing Hormone) OR (Gn-RH) OR (Gonadoliberin) OR (LHFSH Releasing Hormone) OR (Releasing Hormone, LHFSH) OR (LH-RH) OR (LFRH) OR (LH-Releasing Hormone) OR (LH Releasing Hormone) OR (LH-FSH Releasing Hormone) OR (LH FSH Releasing Hormone) OR (LHFSHRH) OR (LHRH) OR (Luliberin) OR (Gonadorelin) OR (Luteinizing Hormone-Releasing Hormone) OR (Luteinizing Hormone Releasing Hormone) OR (FSH-Releasing Hormone) OR (FSH Releasing Hormone) OR (GnRH) OR (Factrel) OR (Cystorelin) OR (Gonadorelin Hydrochloride) OR (Kryptocur) OR (Dirigestran) OR (Gonadorelin Acetate) OR (6 leucine 10 deglycine gonadotropin releasing factor ethylamide) OR ([6 leucine 10 deglycine] gonadotropin releasing factor ethylamide) OR (ay 24, 031) OR (ay 24031) OR (ay24, 031) OR (ay24031) OR (cryptocur) OR (factrel) OR (fertagyl) OR (fertiral) OR (gn rh) OR (gnrh) OR (gnrh serono) OR (gonadoliberin) OR (gonadorelin hydrochloride) OR (gonadotrophin releasing factor) OR (gonadotrophin releasing hormone) OR (gonadotropin release factor) OR (gonadotropin releasing factor) OR (gonadotropin releasing hormone) OR (gonadotropin-releasing hormone) OR (hoe 471) OR (hoe471) OR (hoechst 471) OR (ici 88262) OR (ici88262) OR (kryptocur) OR (lh releasing hormone) OR (lhrf) OR (LHRH) OR (lrh) OR (lrh luteinising hormone releasing factor) OR (lrh luteinizing hormone releasing factor) OR (luforan) OR (luliberin) OR (luliberine) OR (lutal) OR (lutamin) OR (luteinising hormone release factor) OR (luteinising hormone releasing factor) OR (luteinising hormone releasing hormone) OR (luteinizing hormone release factor) OR (luteinizing hormone releasing factor) OR (luteinizing hormone releasing hormone) OR (pulstim) OR (relisorm)) | 45,221 |
| #3 | TI=((Orchiectomies) OR (Orchidectomy) OR (Orchidectomies) OR (Castration, Male) OR (Castrations, Male) OR (Male Castration) OR (Male Castrations) OR (orchectomy) OR (orcheotomy) OR (orchidectomy) OR (testectomy)) OR AK=((Orchiectomies) OR (Orchidectomy) OR (Orchidectomies) OR (Castration, Male) OR (Castrations, Male) OR (Male Castration) OR (Male Castrations) OR (orchectomy) OR (orcheotomy) OR (orchidectomy) OR (testectomy)) OR AB=((Orchiectomies) OR (Orchidectomy) OR (Orchidectomies) OR (Castration, Male) OR (Castrations, Male) OR (Male Castration) OR (Male Castrations) OR (orchectomy) OR (orcheotomy) OR (orchidectomy) OR (testectomy)) | 5,845 |
| #4 | #1 AND #2 AND #3 | 118 |
