# Supplementary material for: Luteinizing hormone-releasing hormone agonists versus orchiectomy in the treatment of prostate cancer: A systematic review
Source: Front Endocrinol (Lausanne). 2023 Feb 6;14:1131715. doi: 10.3389/fendo.2023.1131715 (PMC9939757; doi:10.3389/fendo.2023.1131715)
Supplement: Supplementary file 2 [file Table_2.docx]

Supplementary Material

Luteinizing Hormone-Releasing Hormone Agonists versus Orchiectomy in the treatment of prostate cancer: A Systematic Review

Xianlu Zhang^1^, Gejun Zhang^1^, Jianfeng Wang^1^, Yanli Wang^2*^

*** Correspondence:** Yanli Wang, yanbao213@163.com

# Supplementary Figures 1

#
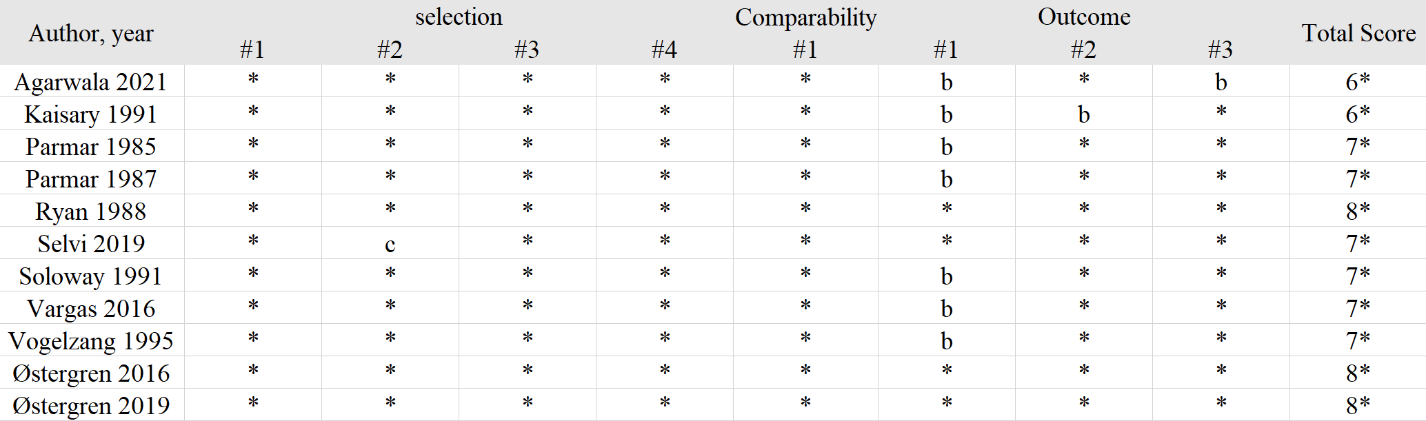
Supplementary Figure 1. Quality assessments of the included studies

# Supplementary Figures 2

**
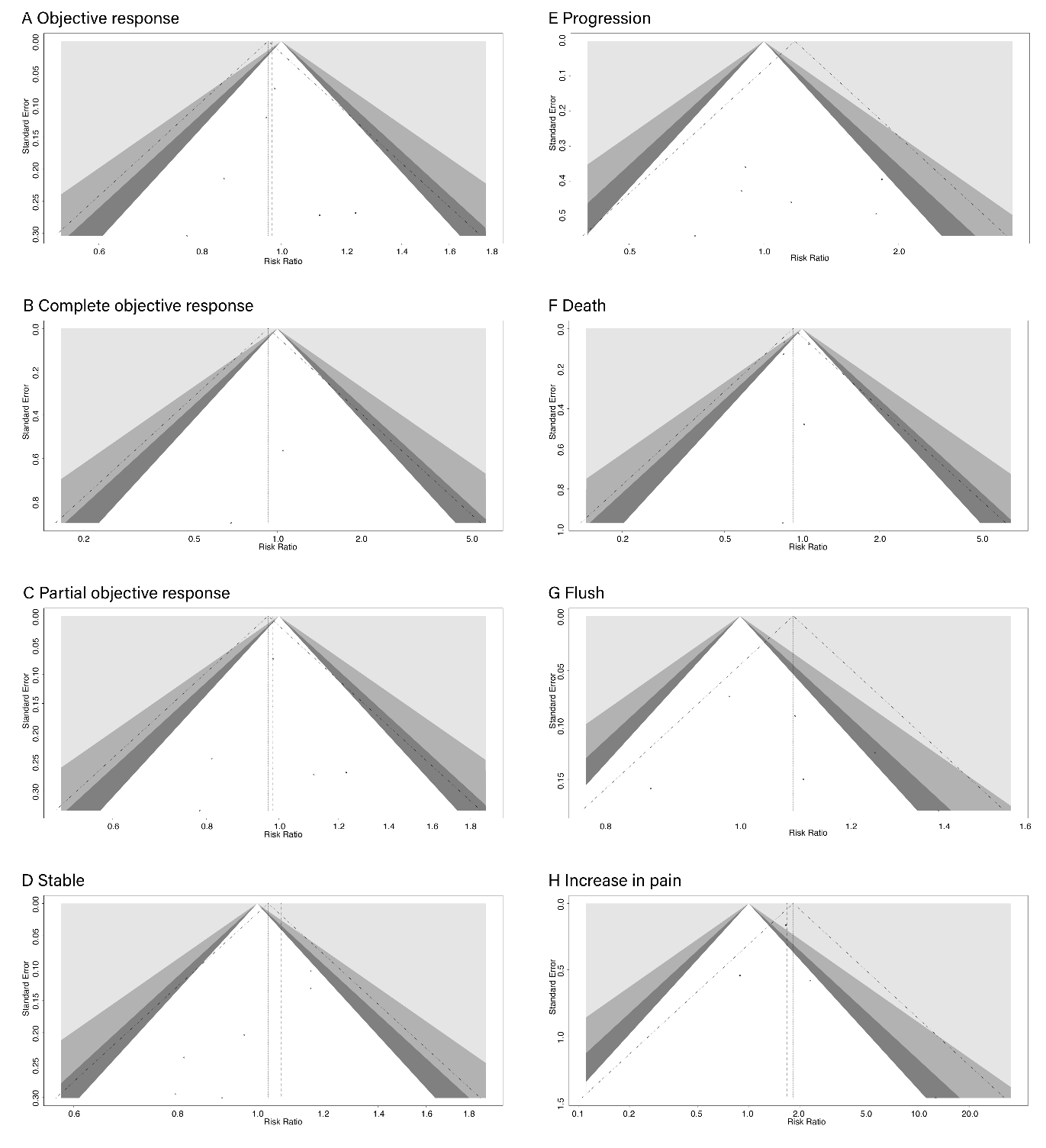
**

**Supplementary Figure 2 Funnel plots of meta-analysis outcomes**

# Supplementary Table 1 Criteria of objective response

| - | Response category | | |
| --- | --- | --- | --- |
|  | Partial response | Stable | Progression |
| Tumor masses | >50% reduction of at least one mass | No increase in size >25%; new lesions | Increase in size >25%; new lesions |
| Acid phosphatase | Normal | Decrease but not to normal | Increase by 50% over previous level |
| Osteoblastic lesions | No progression | Stable | Increase in size and/or no of metastases |
| Osteolytic lesions | Recalcification | Not worse | Increase in size and/or no of metastases |
| Hepatomegaly/abnormal liver function tests | 30% decrease/ 30% decrease | No increase >30%/not worse | Increase in hepatomegaly and/or alkaline phosphatase by 50% |
| Weight, symptoms, performance status | No deterioration | No deterioration | Deterioration |
