# Supplementary material for: Luteinizing hormone-releasing hormone agonists versus orchiectomy in the treatment of prostate cancer: A systematic review
Source: Front Endocrinol (Lausanne). 2023 Feb 6;14:1131715. doi: 10.3389/fendo.2023.1131715 (PMC9939757; doi:10.3389/fendo.2023.1131715)
Supplement: Supplementary file 3 [file Table_3.docx]

Supplementary Material

Luteinizing Hormone-Releasing Hormone Agonists versus Orchiectomy in the treatment of prostate cancer: A Systematic Review

Xianlu Zhang^1^, Gejun Zhang^1^, Jianfeng Wang^1^, Yanli Wang^2*^

*** Correspondence:** Yanli Wang, yanbao213@163.com

# R codes

library("meta")

library("xlsx")

setwd("C:/Users/xianluzhang/Desktop/睾丸切除术和ADT的并发症比较/数据分析")

getwd()

STAdt <- read.xlsx("STA.xlsx", sheetIndex = 2, header = TRUE)

STAdt

STAmt <- metabin(data = STAdt, studlab = STAdt$study,

event.e, n.e, event.c, n.c,

sm = "RR")

STAmt

dev.new()

setEPS()

postscript("RES森林图.eps", width = 30, height = 15)

forest(RESmt, label.e = "MEDI", label.c = "ORCI")

dev.off()

dev.new()

setEPS()

postscript("STA漏斗图.eps", width = 30, height = 15)

cc<-funnel(STAmt,comb.fixed=TRUE, level=0.95, contour=c(0.9, 0.95, 0.99))$col.contour

dev.off()

# Original data

| Objective response | | | | |
| --- | --- | --- | --- | --- |
| study | event.e | n.e | event.c | n.c |
| Kaisary 1991 | 71 | 148 | 72 | 144 |
| Parmar 1985 | 19 | 38 | 13 | 32 |
| Parmar 1987 | 20 | 55 | 16 | 49 |
| Ryan 1988 | 105 | 148 | 104 | 144 |
| Soloway 1991 | 15 | 81 | 20 | 83 |
| Vogelzang 1995 | 30 | 138 | 37 | 145 |
| Complete objective response | | | | |
| study | event.e | n.e | event.c | n.c |
| Ryan 1988 | 0 | 148 | 0 | 144 |
| Soloway 1991 | 2 | 81 | 3 | 83 |
| Vogelzang 1995 | 6 | 138 | 6 | 145 |
| Partial objective response | | | | |
| study | event.e | n.e | event.c | n.c |
| Kaisary 1991 | 11 | 148 | 6 | 144 |
| Parmar 1985 | 5 | 38 | 6 | 32 |
| Parmar 1987 | 9 | 55 | 9 | 49 |
| Ryan 1988 | 17 | 148 | 9 | 144 |
| Soloway 1991 | 9 | 81 | 8 | 83 |
| Vogelzang 1995 | 13 | 138 | 15 | 145 |
| Stable status | | | | |
| study | event.e | n.e | event.c | n.c |
| Kaisary 1991 | 18 | 148 | 22 | 144 |
| Parmar 1985 | 14 | 38 | 13 | 32 |
| Parmar 1987 | 26 | 55 | 24 | 49 |
| Ryan 1988 | 26 | 148 | 31 | 144 |
| Soloway 1991 | 51 | 81 | 45 | 83 |
| Vogelzang 1995 | 84 | 138 | 76 | 145 |
| Progression rate | | | | |
| study | event.e | n.e | event.c | n.c |
| Kaisary 1991 | 11 | 148 | 6 | 144 |
| Parmar 1985 | 5 | 38 | 6 | 32 |
| Parmar 1987 | 9 | 55 | 9 | 49 |
| Ryan 1988 | 17 | 148 | 9 | 144 |
| Soloway 1991 | 9 | 81 | 8 | 83 |
| Vogelzang 1995 | 13 | 138 | 15 | 145 |
| Death rate | | | | |
| study | event.e | n.e | event.c | n.c |
| Kaisary 1991 | 102 | 148 | 116 | 144 |
| Parmar 1985 | 2 | 38 | 2 | 32 |
| Parmar 1987 | 8 | 55 | 7 | 49 |
| Ryan 1988 | 62 | 148 | 71 | 144 |
| Vogelzang 1995 | 98 | 138 | 97 | 145 |
| Flush rate | | | | |
| study | event.e | n.e | event.c | n.c |
| Kaisary 1991 | 96 | 152 | 94 | 163 |
| Parmar 1985 | 29 | 38 | 22 | 32 |
| Parmar 1987 | 31 | 55 | 32 | 49 |
| Ryan 1988 | 105 | 148 | 104 | 144 |
| Soloway 1991 | 42 | 81 | 31 | 83 |
| Vogelzang 1995 | 73 | 135 | 61 | 141 |
| Increase in pain rate | | | | |
| study | event.e | n.e | event.c | n.c |
| Kaisary 1991 | 6 | 148 | 0 | 144 |
| Parmar 1987 | 6 | 55 | 6 | 49 |
| Ryan 1988 | 6 | 148 | 0 | 144 |
| Soloway 1991 | 9 | 81 | 4 | 83 |
| Vogelzang 1995 | 63 | 138 | 40 | 145 |

The original data of meta-analysis. ‘event.e’ indicates LHRH options, ‘event.c’ indicates orchiectomy options.
